# Projected spatiotemporal shifts in forest community suitability in the northeastern Qinghai-Tibet Plateau under climate change

**DOI:** 10.3389/fpls.2026.1877886

**Published:** 2026-07-29

**Authors:** Long Chen, Xiaohua Gou, Fen Zhang, Zibo Wang, Dingcai Yin

**Affiliations:** 1 Key Laboratory of Western China’s Environmental Systems with the Ministry of Education, College of Earth and Environmental Sciences, Lanzhou University, Lanzhou, China; 2 Gansu Natural Energy Research Institute, Gansu Academy of Sciences, Lanzhou, China; 3 Gansu Liancheng Forest Ecosystem Field Observation and Research Station, Lanzhou University, Lanzhou, China; 4 Academy of Plateau Science and Sustainability, Qinghai Normal University, Xining, China; 5 School of Earth Sciences and Spatial Information Engineering, Hunan University of Science and Technology, Xiangtan, China

**Keywords:** climate change, forest community, Qinghai-Tibet Plateau, random forest model, spatiotemporal dynamics, suitable habitats

## Abstract

The northeastern Qinghai-Tibet Plateau (QTP) constitutes a critical ecological security barrier and an important terrestrial carbon sink in China. Forest community in this region plays an indispensable role in water conservation, soil retention, and carbon sequestration. However, it remains unclear how ongoing climate change is expected to alter the distribution patterns of forest community. Here, using 2,892 forest community occurrence records, we developed random forest (RF) models to project changes in climatically suitable habitats for different forest community types under two climate scenarios: a sustainability pathway (SSP1-2.6) and a regional rivalry pathway (SSP3-7.0). Our results indicate that the total area of climatically suitable forest habitats is projected to expand substantially under both scenarios, with the overall suitable area expanding by approximately twofold compared with the current period. Responses, however, vary among community types, with evergreen coniferous forest (ECF) showing the most pronounced increase in suitable habitats, whereas the expansion of deciduous broadleaf forest (DBF) is relatively limited. Spatially, forest suitable habitats are projected to expand primarily in the eastern Qilian Mountains and northern Gannan Plateau, which represent potential hotspots for forest expansion. In contrast, areas currently suitable for ECF on the northern slope of the Qilian Mountains and for DBF in the southern mountainous regions of the Gannan Plateau are projected to become unsuitable under climate conditions, suggesting a redistribution of forest community along regional climatic gradients. These findings highlight contrasting climatic sensitivities among forest formations and underscore the urgency of incorporating climatic suitability into forest conservation and management strategies.

## 1 Introduction

Global climate warming has been widely recognized, and the Qinghai-Tibet Plateau (QTP) has exhibited an amplified response compared with the global average (Wang et al., 2025; Yang et al., 2025; Latif et al., 2019). Over the past five decades, warming rates across the QTP have reached approximately 0.3-0.4°C per decade, exceeding twice the global mean rate and marking the warmest period in the past two millennia (Chen et al., 2015; Yao, 2019). Climate change is widely acknowledged as a dominant driver of vegetation dynamics in ecologically vulnerable regions (Pu et al., 2025; Li et al., 2023b). Rising temperatures and shifting precipitation regimes influence plant physiology, phenology, and species distributions (Su et al., 2022; Wang et al., 2012; Gao et al., 2018), thereby reshaping dominant vegetation types and altering ecosystem functioning (Piao et al., 2011).

The northeastern QTP, located at the transitional zone between the first and second geomorphological steps of China, represents a convergence area of the Qinghai-Tibet, Mongolian, and Loess plateaus. This region serves as a key ecological security barrier for northern China (Sun et al., 2012; Chen et al., 2021; Liu et al., 2019). Forests in this region provide multiple ecosystem services, including water conservation, soil retention, and carbon storage (Zhao et al., 2024; Liang et al., 2022; Ma et al., 2021). Owing to complex topographic and climatic gradients, forest community exhibits pronounced compositional heterogeneity, ranging from cold- and drought-adapted evergreen coniferous forest to relatively warm- and moisture-favoring deciduous broadleaf forest (Chen et al., 2014). Against the backdrop of global climate change, the forest community in the region is undergoing unprecedented transformations (Gao et al., 2021; Ni, 2000; Wijenayake et al., 2025). For instance, some studies indicate that the distribution boundaries of certain tree species are migrating to higher elevations due to warming climates, while species adapted to colder climates face survival threats (Zhang et al., 2023a; Lu et al., 2019). However, the responses of forest growth to warming are not uniform; some studies have reported relatively consistent growth-climate relationships along an elevation gradient (Li et al., 2020), whereas others have documented contrasting patterns, with reduced growth at lower elevations but enhanced growth at higher elevations (Shi et al., 2021; Panthi et al., 2020). These discrepancies may partly reflect differences in the magnitude of regional climate warming, but more importantly, they are likely associated with varying response strategies among different forest community types (Lenoir et al., 2010; Zhou et al., 2025).

Understanding whether climatic conditions will favor coniferous or broadleaf forests is essential for effective forest management and restoration planning. Despite extensive research on vegetation dynamics in the QTP, projections at the vegetation formation level remain limited (Su et al., 2020; Ni, 2000). Machine-learning approaches, particularly the random forest (RF) algorithm, provide powerful tools for modeling nonlinear relationships between plant distributions and environmental variables (Cheng et al., 2023; Zhang et al., 2025). RF integrates bootstrap aggregation and random feature selection, offering high predictive accuracy and robustness under complex ecological conditions (Rather et al., 2020; Bhuyan et al., 2025). By combining field survey data with environmental predictors, RF enables spatially explicit projections of climatically suitable habitats, thereby facilitating the evaluation of differential responses among forest community types (Sun et al., 2020). The method maintains high predictive accuracy and stability, and its outputs can be readily visualized in a Geographic Information System (GIS) (Moreno et al., 2011).

This study integrates 2,892 forest community occurrence records with climatic and terrain variables to project habitat suitability under different climate scenarios. Specifically, we aim to: (1) project changes in forest community distribution across different vegetation classification units in the northeastern QTP; (2) identify the spatial locations of suitable habitats for different forest types; (3) identify the primary environmental drivers governing their distributions. By clarifying contrasting responses among forest formations, this study provides a scientific basis for adaptive forest management and conservation planning in high-elevation transitional regions.

## 2 Materials and methods

### 2.1 Study area and field survey data

The study area encompasses the Qilian Mountains and the Gannan Plateau, where forest community is primarily distributed at elevations between approximately 2200 m and 4000 m. Forest community composition differs markedly between the two subregions. In the Qilian Mountains, forest community structure is relatively simple and is dominated by evergreen coniferous forest. Shady slopes are primarily occupied by *Picea crassifolia*, whereas sunny slopes are dominated by *Juniperus przewalskii*. Only limited and fragmented patches of *Picea wilsonii* and *Pinus tabuliformis* occur in the easternmost part of the range. In contrast, the Gannan Plateau represents a biogeographical transition zone where cold-temperate coniferous forest extend southward from the northern Plateau and intersect with deciduous broadleaf forest expanding northward from lower latitudes. Consequently, forest community in this region exhibits higher compositional diversity and stronger transitional characteristics.

Extensive field surveys were conducted across the study area to systematically document forest community distributions. Occurrence records were collected using LiVegetation, a mobile application designed to support standardized and georeferenced forest community sampling (Jin et al., 2021). To ensure ecological representativeness, sampling points were restricted to natural vegetation sites with minimal human disturbance, and each record was required to accurately represent the corresponding vegetation alliance (Su et al., 2020). During the field surveys, both the vegetation alliance type and the precise geographic coordinates of each sampling point were recorded.

**Figure 1:**
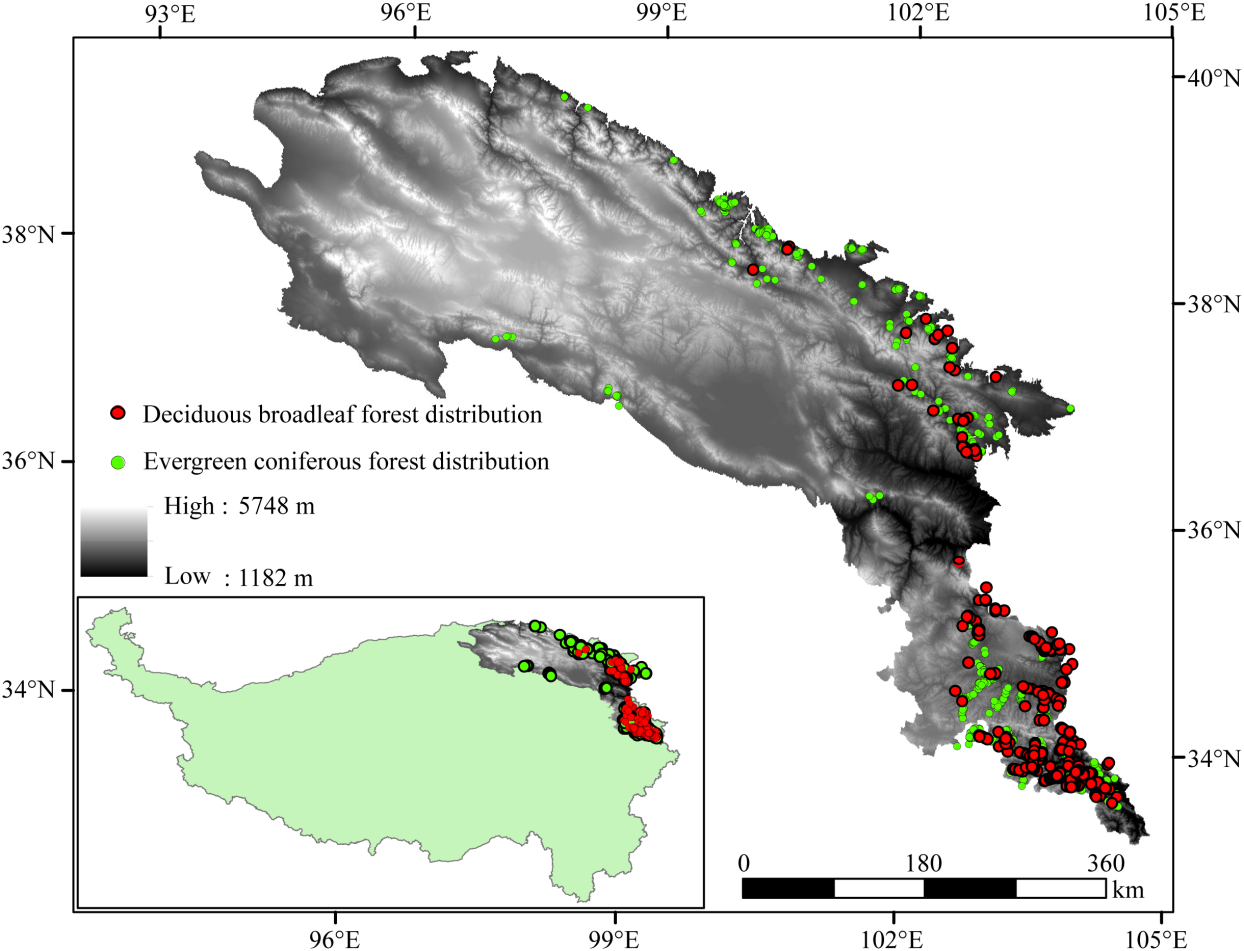
Distributions of study area and field survey points.

A total of 2,892 forest occurrence records were compiled in this study, including 1,920 records of evergreen coniferous forest (ECF) and 696 records of deciduous broadleaf forest (DBF). In addition, 276 records represented deciduous coniferous forests dominated by *Larix potaninii*, as well as some mixed coniferous-broadleaf forests, owing to their extremely limited distribution and small sample size, these forest community types were not modeled separately but were instead incorporated into the overall forest analysis (**Figure 1**). Vegetation classification followed the China Vegetation Classification System (China-VCS) (Guo et al., 2020).

### 2.2 Selection of environmental variables

We selected 19 bioclimatic variables and three terrain variables to characterize temperature, precipitation, and topographic conditions (**Table 1**). Climatic data for the baseline period (1970–2000) and future projections (2041–2060 and 2061-2080) were obtained from WorldClim (https://worldclim.org/). Future climate data were obtained under two Shared Socioeconomic Pathways (SSPs): SSP1-2.6, representing a sustainability pathway, and SSP3-7.0, representing a regional rivalry pathway (IPCC, 2021). We used five different global circulation models (GCMs) from the sixth coupled model intercomparison project (CMIP6), including ACCESS-CM2, BCC-CSM2-MR, CMCC-ESM2, INM-CM5-0, and MRI-ESM2-0 (Shi et al., 2023; Li et al., 2023a). Instead of averaging predictions from multiple independently simulated models, we generated climate projections using a single RF framework driven by ensemble-mean climate variables derived from the five GCMs (Manrique-Ascencio et al., 2024; Van Tiel et al., 2024). Terrain variables, including elevation, slope, and aspect, were extracted from the ASTER GDEM dataset (METI and NASA, 2019). All environmental layers were resampled to a consistent spatial resolution of 30 arc seconds (approximately 1 km).

**Table 1:** Environmental variables used in the models.

Types	Variables	Descriptor
Temperature	Bio1	Annual mean temperature (°C)
	Bio2	Mean diurnal temperature range (°C)
	Bio3	Isothermality (Bio2/Bio7×100)
	Bio4	Temperature seasonality (standard deviation×100)
	Bio5	Maximum temperature of warmest month (°C)
	Bio6	Minimum temperature of coldest month (°C)
	Bio7	Temperature annual range (Bio5-Bio6)
	Bio8	Mean temperature of wettest quarter (°C)
	Bio9	Mean temperature of driest quarter (°C)
	Bio10	Mean temperature of warmest quarter (°C)
	Bio11	Mean temperature of coldest quarter (°C)
Precipitation	Bio12	Annual precipitation (mm)
	Bio13	Precipitation of wettest month (mm)
	Bio14	Precipitation of driest month (mm)
	Bio15	Precipitation seasonality (coefficient of variation)
	Bio16	Precipitation of wettest quarter (mm)
	Bio17	Precipitation of driest quarter (mm)
	Bio18	Precipitation of warmest quarter (mm)
	Bio19	Precipitation of coldest quarter (mm)
Terrain	Elevation	Elevation (m)
	Aspect	Aspect (°)
	Slope	Slope (°)

### 2.3 Random forest model optimization, simulation and evaluation

Random forest (RF) is an ensemble learning method that integrates multiple decision trees constructed using bootstrap samples and randomly selected subsets of environmental predictors, enabling robust predictions of nonlinear relationships between forest distributions and environmental variables (Breiman, 2001). We developed RF classification models for different levels of forest community types to predict current and future distributions of climatically suitable habitats. Environmental covariates were extracted for each presence record, and pseudo-absence background points were randomly sampled across the study area at a 5:1 ratio relative to presence records. All records containing missing environmental values were discarded prior to downstream analysis.

To avoid overly optimistic model evaluation caused by spatial autocorrelation among occurrence points and ensure the spatial independence of training and validation datasets—an essential prerequisite for reliable spatial predictions—we adopted a spatial block cross-validation framework instead of simple random data splitting. This spatial partitioning strategy strictly separates geographically adjacent samples into distinct folds, eliminating spatial leakage between training and testing subsets and substantially improving the credibility of subsequent habitat suitability projections. The full dataset was divided into five spatially independent folds, with each fold serving as an independent test set once, while the remaining four folds were combined for model training. The RF classifier was constructed with 500 decision trees, and the number of variables randomly selected at each tree split was defined as the square root of the total number of environmental covariates. The relative importance of variables was quantified via the mean decrease in Gini impurity, which reflects the contribution of each variable to discriminating suitable and unsuitable habitats (Allouche et al., 2006; Lobo et al., 2008; Beale and Lennon, 2012). Model predictive performance was evaluated on spatially independent test folds using two robust complementary metrics: the true skill statistic (TSS) and Cohen’s Kappa coefficient. Higher values for TSS and Kappa indicate superior discriminatory power and more reliable habitat suitability predictions.

To convert continuous habitat suitability probabilities (ranging from 0 to 1) into binary maps of suitable and unsuitable habitat, the optimal threshold was determined by maximizing the true skill statistic (TSS) (Liu et al., 2016). Grid cells with predicted suitability values greater than or equal to this threshold were classified as suitable habitat, whereas those below the threshold were classified as unsuitable. All statistical and spatial analyses were conducted in R (version 4.5.2).

### 2.4 Spatiotemporal distribution and mapping of suitable habitat

Based on the outputs of the RF models, habitat suitability was reclassified into binary maps using ArcGIS (version 10.8), with two categories representing suitable and unsuitable habitats. This reclassification generated binary habitat suitability matrices under both current and future climate scenarios. The future periods were defined as the near-term 2050s (2041–2060) and the long-term 2070s (2061–2080). Under climate change scenarios, spatial dynamics of suitable habitats were characterized by three types of changes: expansion, contraction, and stability. In addition, the geometric centroid of suitable habitat patches was calculated using the ArcMap tool in ArcGIS to represent the distribution center of suitable areas, thereby reflecting the overall spatial shift in potential habitats of different forest types (Zhao et al., 2021).

## 3 Results

### 3.1 Model robustness assessment and determination of binary classification thresholds

All RF models demonstrated excellent predictive performance (**Table 2**). The forest model (Model 1) achieved a TSS value of 0.894 and a Kappa coefficient of 0.870, demonstrating a high level of agreement between model predictions and observed occurrences records. The optimal threshold for binary classification was identified as 0.405 based on the maximum TSS criterion, which was subsequently used to convert continuous suitability outputs into binary suitable and unsuitable habitat maps.

**Table 2:** Estimated values of models.

Model	Type	Occurrence records	TSS	Kappa	TSS-maximized threshold
Model 1	Forest	2,892	0.894	0.870	0.405
Model 2	Evergreen coniferous forest	1,920	0.871	0.839	0.405
Model 3	Deciduous broadleaf forest	696	0.908	0.906	0.400

For models developed for specific forest vegetation formations, the ECF model (Model 2) showed good predictive capability, with a TSS value of 0.871 and a Kappa coefficient of 0.839. The TSS-maximizing threshold was also determined to be 0.405, indicating a stable classification boundary for identifying evergreen coniferous forest areas. The DBF model (Model 3) exhibited the highest predictive accuracy among all models, achieving a TSS value of 0.908 and a Kappa coefficient of 0.906. The corresponding optimal threshold was 0.400, suggesting a slightly lower probability cutoff for separating suitable and unsuitable habitats compared with the other models.

### 3.2 Suitable habitat distribution and area under climate scenarios

#### 3.2.1 Forest vegetation formation group

**Figure 2:**
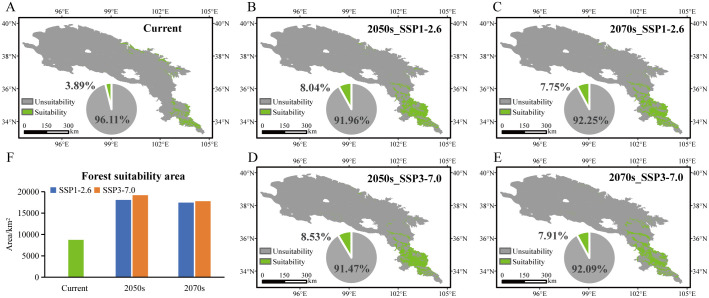
Current and future suitable habitat distribution and area of forest vegetation formation group. Note: Panels show the distribution and area under different periods and scenarios: **(A)** current, **(B)** 2050s_SSP1-2.6, **(C)** 2070s_SSP1-2.6, **(D)** 2050s_SSP3-7.0, **(E)** 2070s_SSP3-7.0, **(F)** suitability area change under different periods and scenarios.

Under current climatic conditions, forest community does not represent a dominant vegetation type within the study area, with suitable habitats accounting for only 3.89% of the total area according to the RF model (**Figure 2A**). The current forest community distribution is primarily characterized by ECF, which are mainly concentrated in the northern part of the study area and the high-elevation mountainous regions in the south. DBF constitutes a smaller proportion of the forest community and are mainly distributed in the lower-elevation mountainous areas of the southeastern region. Overall, the existing forest community types exhibit strong adaptation to cold and arid alpine climate conditions of the plateau environment.

Compared with the current period, the proportion of suitable habitats increased under both climate scenarios. Under SSP1-2.6, suitable habitats accounted for 8.04% and 7.75% of the study area in the 2050s and 2070s, respectively (**Figures 2B, C**). The corresponding proportions under SSP3-7.0 were 8.53% and 7.91% in the 2050s and 2070s, respectively (**Figures 2D, E**). The suitable habitat extent increased substantially from approximately 8,000 km² under current conditions to approximately 17,000–18,000 km² during future periods. Under SSP1-2.6, suitable habitats covered approximately 17,000 km² in the 2050s and increased slightly to 17,500 km² in the 2070s. The corresponding values under SSP3-7.0 were approximately 18,000 km² in both the 2050s and 2070s (**Figure 2F**).

#### 3.2.2 Evergreen coniferous forest

**Figure 3:**
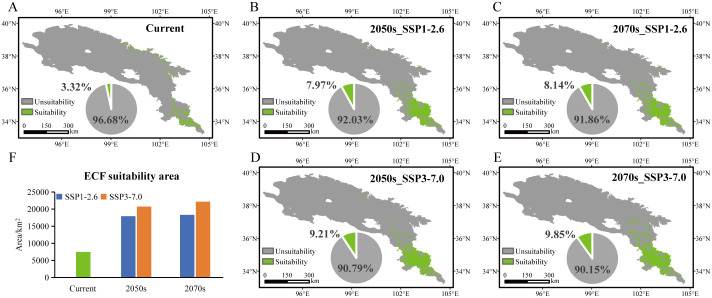
Current and future suitable habitat distribution and area of evergreen coniferous forest. Panels show the distribution and area under different periods and scenarios: **(A)** current, **(B)** 2050s_SSP1-2.6, **(C)** 2070s_SSP1-2.6, **(D)** 2050s_SSP3-7.0, **(E)** 2070s_SSP3-7.0, **(F)** suitability area change under different periods and scenarios.

ECF represents the most widely distributed forest community type in the study area. Under current climatic conditions, suitable habitats account for 3.32% of the total study area and are mainly distributed in the eastern and southeastern regions (**Figure 3A**), which generally correspond to its current distribution range. The dominant tree species of ECF are mainly cold- and drought-tolerant coniferous species from the genera *Picea* and *Juniperus*, including *Picea crassifolia*, *Picea asperata*, *Picea wilsonii*, *Juniperus przewalskii*, and *Juniperus tibetica*, among others.

Under climate scenarios, the extent of suitable habitat is projected to expand relative to the current period. In the SSP1-2.6 scenario, suitable habitat increases to 7.97% by the 2050s and rises slightly further to 8.14% by the 2070s (**Figures 3B, C**). Under the SSP3-7.0 scenario, a more pronounced expansion is observed, with suitable habitat reaching 9.21% in the 2050s and 9.85% in the 2070s (**Figures 3D, E**). The suitable habitat area of ECF showed a similar increasing trend under future periods and scenarios (**Figure 3F**). Compared with the current period, the suitable habitat area increased under both scenarios. Under SSP1-2.6, the suitable habitat extent reached approximately 18,000 km² in the 2050s and remained relatively stable in the 2070s. In comparison, SSP3-7.0 resulted in a larger suitable habitat extent, increasing to approximately 20,000 km² in the 2050s and further expanding to approximately 21,000 km² by the 2070s.

#### 3.2.3 Deciduous broadleaf forest

**Figure 4:**
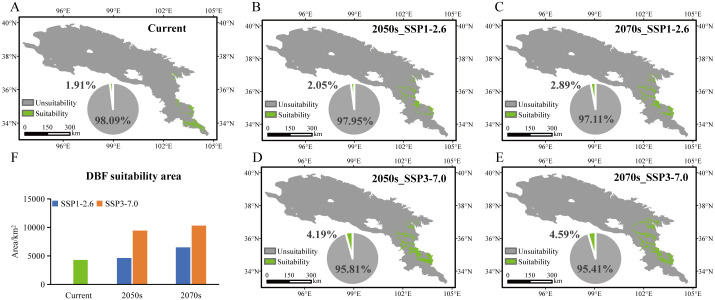
Current and future suitable habitat distribution and area of deciduous broadleaf forest. Panels show the distribution and area under different periods and scenarios: **(A)** current, **(B)** 2050s_SSP1-2.6, **(C)** 2070s_SSP1-2.6, **(D)** 2050s_SSP3-7.0, **(E)** 2070s_SSP3-7.0, **(F)** suitability area change under different periods and scenarios.

DBF is not the dominant forest community type in any period, with consistently low proportional coverage and a relatively limited spatial distribution across all time slices. Under current conditions, habitat suitability is very low, accounting for only 1.91% of the total area (**Figure 4A**), and the distribution is highly discontinuous, appearing only in small, scattered patches. The dominant tree species of DBF include *Betula platyphylla*, *Populus davidiana*, and *Betula albosinensis*, among others.

In the climate projections, a gradual but uneven increase in suitable habitat is observed. Under the SSP1-2.6 scenario, suitability rises slightly to 2.05% in the 2050s (**Figure 4B**) and further to 2.89% in the 2070s (**Figure 4C**), indicating a relatively restrained response under a low-emission pathway. By contrast, the SSP3-7.0 scenario shows a clearer upward shift, with suitable area increasing to 4.19% in the 2050s (**Figure 4D**) and reaching 4.59% in the 2070s (**Figure 4E**), suggesting stronger climate sensitivity under higher forcing conditions. The overall comparison of area changes (**Figure 4F**) further highlights the divergence between scenarios, with SSP3-7.0 consistently producing larger suitable habitat extents than SSP1-2.6 across future periods. Nevertheless, even under the most extreme scenario, DBF remains a non-dominant vegetation type in the region, and its spatial expansion is still relatively constrained and fragmented rather than forming extensive continuous distributions.

### 3.3 Spatial redistribution of suitable habitats

**Figure 5:**
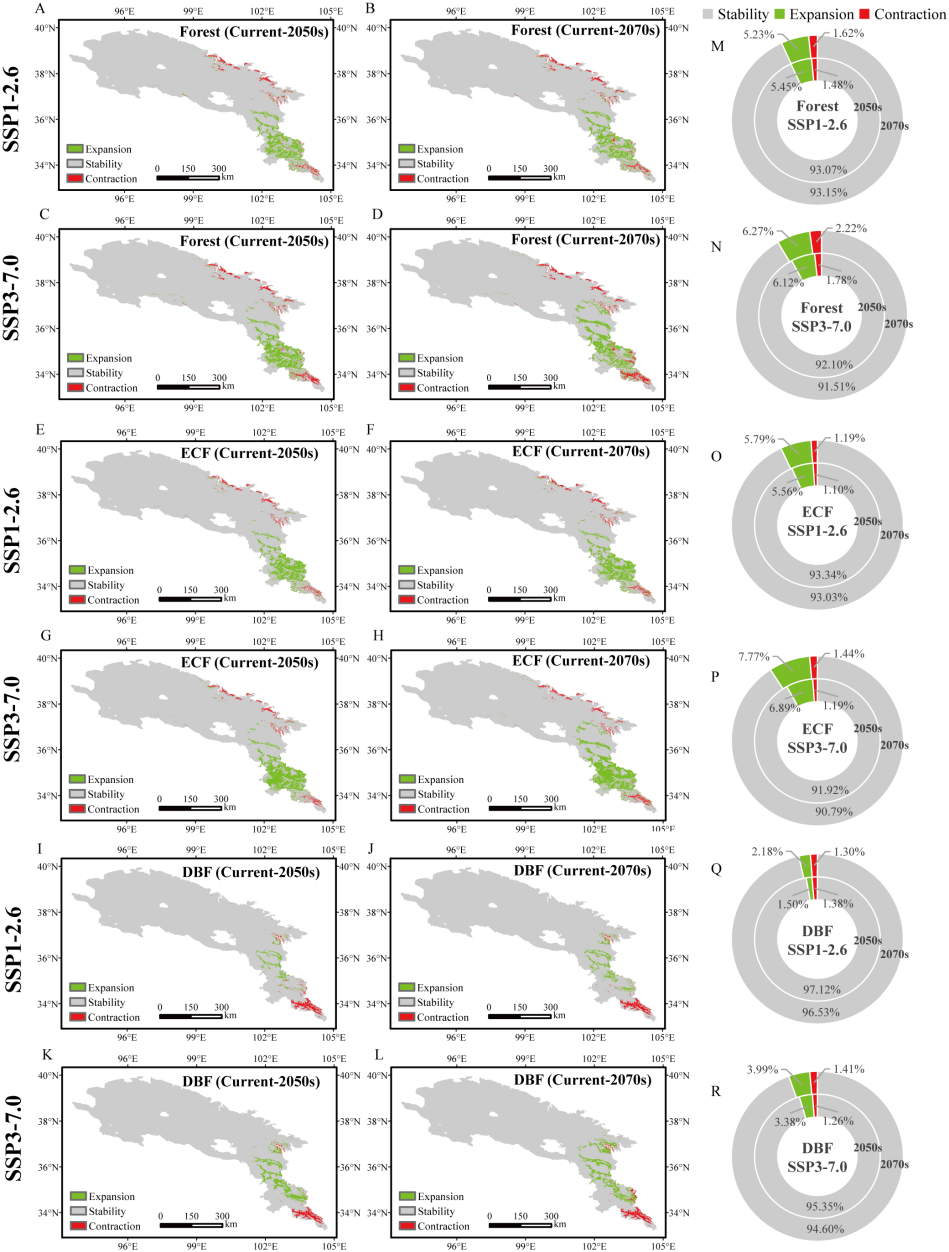
Spatiotemporal variations of suitable habitats for forest, ECF and DBF under SSP1-2.6 and SSP3-7.0 scenarios in the 2050s and 2070s. **(A, C, E, G, I, K)** Habitat change from current to the 2050s; **(B, D, F, H, J, L)** Habitat change from current to the 2070s. Circular proportion diagrams **(M–R)** quantify the percentage of stable, expanded and contracted suitable habitat areas for each forest type and period.

**Figure 6:**
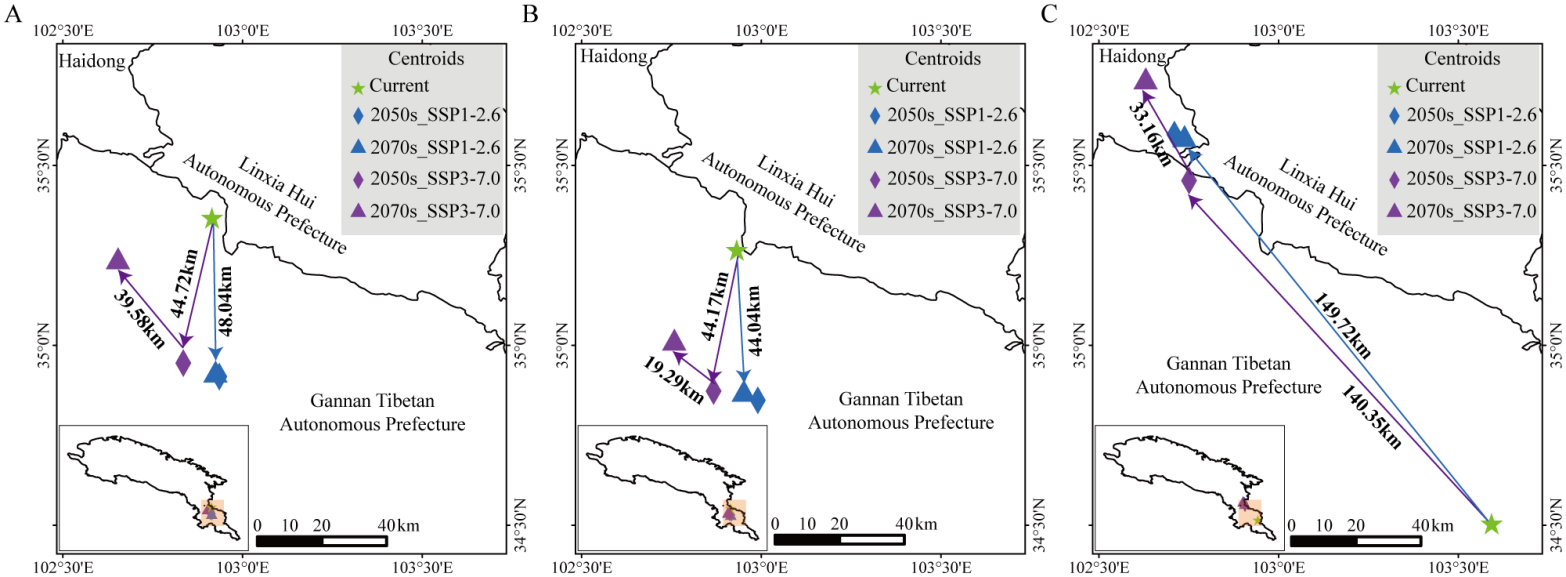
Centroids of suitable habitat distribution for different forest types across different periods and climate scenarios: **(A)** forest, **(B)** ECF, **(C)** DBF.

In addition to changes in total area, suitable habitats exhibit pronounced spatial redistribution patterns. At the vegetation formation group level, under both climate scenarios, the areas currently suitable for forest in the central Qilian Mountains and southern Gannan Plateau are projected to undergo contraction, while suitable forest expansion will be concentrated in the eastern Qilian Mountains and northern Gannan Plateau, and this trend remains consistent across different periods. (**Figures 5A–D**). From the 2050s to the 2070s, SSP1-2.6 shows a slight increase in stable habitat coverage, whereas SSP3-7.0 exhibits the opposite pattern, with more extensive habitat expansion and contraction but lower stability relative to SSP1-2.6 (**Figures 5M, N**). Before the 2050s, the geometric centroid of suitable distribution areas consistently shifted southward by more than 40 km. After the 2050s, however, divergent patterns emerged across different climate scenarios. Under SSP1-2.6, the centroid showed only minor, statistically negligible movement, whereas under SSP3-7.0, it shifted approximately 40 km toward the northwest (**Figure 6A**).

For ECF, a similar temporal and spatial pattern to that of the forest vegetation formation group is observed. The eastern Qilian Mountains and the central-northern part of the Gannan Plateau are identified as the primary regions of future ECF expansion (**Figures 5E–H**). The most pronounced expansion occurs from the present to the 2050s, whereas from the 2050s to the 2070s, the increase in area becomes limited (**Figures 5O, P**). In addition, areas currently suitable for ECF distribution on the northern slopes of the Qilian Mountains and in the southern Gannan Plateau are projected to become unsuitable in the future. Accordingly, the centroid of the suitable distribution area also exhibited a trend similar to that observed for forest vegetation formation group (**Figure 6B**).

In the future, the expansion of suitable distribution areas for DBF will be primarily concentrated in the eastern Qilian Mountains and the northern Gannan Plateau, while the currently dominant suitable areas in the southern Gannan Plateau are projected to become unsuitable (**Figures 5I–L**). Overall, the centroid of suitable distribution areas exhibited a clear northward shift (**Figure 6C**). As time progresses, the extent of habitat expansion increases, and this trend is consistent across different climate scenarios (**Figures 5Q, R**).

### 3.4 Major environmental drivers of forest distribution

The environmental drivers of forest community distribution vary substantially across hierarchical levels and forest types. At the vegetation formation group level, forest distribution is jointly regulated by moisture and thermal conditions. RF model results indicate that precipitation seasonality is the most influential predictor (14.35%), followed by mean temperature of coldest quarter (11.04%) and annual precipitation (9.91%). Terrain variables play a secondary but non-negligible role, with elevation contributing 6.82%, while slope and aspect show relatively low importance (<2%). (**Table 3**).

**Table 3:** Contributions of environmental variables in the models.

Environment factors		Contribution rate / %		
Types	Descriptions	Forest	ECF	DBF
Precipitation	Precipitation seasonality	**14.35**	**11.99**	**16.10**
	Annual precipitation	9.91	**10.36**	7.16
	Precipitation of wettest quarter	4.47	3.96	3.74
	Precipitation of warmest quarter	3.01	3.24	3.10
	Precipitation of wettest month	2.66	2.76	3.81
	Precipitation of driest quarter	1.14	1.58	0.81
	Precipitation of coldest quarter	1.24	1.54	0.75
	Precipitation of driest month	0.32	0.52	0.22
Temperature	Mean temperature of coldest quarter	**11.04**	9.72	**11.07**
	Minimum temperature of coldest month	7.80	6.99	**13.44**
	Annual mean temperature	4.79	5.15	9.06
	Temperature seasonality	6.15	5.34	3.83
	Temperature annual range	4.48	4.64	3.96
	Mean temperature of driest quarter	3.68	3.51	3.36
	Mean diurnal temperature range	3.32	3.97	3.42
	Mean temperature of wettest quarter	3.10	3.73	3.74
	Isothermality	2.95	3.57	1.38
	Mean temperature of warmest quarter	2.92	3.15	2.83
	Maximum temperature of warmest month	2.25	2.72	2.84
Terrain	Elevation	6.82	7.50	3.02
	Slope	1.92	2.36	1.35
	Aspect	1.67	1.71	1.00

ECF, evergreen coniferous forest; DBF, deciduous broadleaf forest.

Values with a contribution rate greater than 10 are marked in bold.

At the vegetation formation level, the relative importance of environmental variables differs markedly among forest types. ECF shows a relatively balanced control by precipitation, temperature, and topography. Precipitation seasonality (11.99%) and annual precipitation (10.36%) are the two most important drivers, closely followed by mean temperature of the coldest quarter (9.72%). Notably, elevation exhibits a comparatively higher contribution (7.50%) in ECF than in other forest types. In addition, minimum temperature of the coldest month (6.99%) further indicates the sensitivity of ECF to extreme winter temperature conditions. In contrast, DBF is more strongly regulated by thermal constraints. Precipitation seasonality remains the single most important predictor (16.10%), but the minimum temperature of the coldest month (13.44%) and annual mean temperature (9.06%) together indicate a pronounced dependence on low-temperature thresholds and overall thermal regime (**Table 3**). Compared with ECF, DBF shows weaker dependence on precipitation-related variables, suggesting that its distribution is more tightly constrained by temperature extremes than by moisture availability.

## 4 Discussion

### 4.1 Model robustness and predictive reliability

All RF models exhibited high predictive accuracy, demonstrating their robustness in capturing the relationships between forest community distributions and environmental variables (Rather et al., 2020). Although model performance can be influenced by sample size, the consistently high TSS and Kappa values suggest that predictive reliability was not substantially constrained by differences in occurrence records among forest types. Notably, the DBF model achieved the highest evaluation metrics despite having fewer occurrence records than ECF model (**Table 2**). This pattern may be attributed to stronger climatic constraints on DBF distribution, resulting in clearer environmental thresholds between suitable and unsuitable habitats. In contrast, ECF occupies broader elevational gradients and may exhibit greater ecological flexibility, potentially increasing classification uncertainty. Overall, the high predictive performance supports the reliability of projected spatial redistribution patterns under climate scenarios.

### 4.2 Differential responses of forest distribution across hierarchical levels

Overall forest suitability is projected to increase under climate scenarios (**Figures 2**–**4**), which is consistent with long-term remotely sensed observations and previous model-based projections showing widespread vegetation greening across the QTP (Cui and Graf, 2009; Li et al., 2023b; Wang et al., 2023; Ni, 2000; Li et al., 2024). This overall expansion suggests that regional climate change may alleviate current climatic limitations, particularly thermal constraints in cold alpine environments. The consistent increase in suitable habitat under both SSP1-2.6 and SSP3-7.0 scenarios indicates that warming temperatures may create more favorable conditions for forest establishment and persistence.

However, responses at the vegetation formation level differ among forest types (**Figures 3**, **4**). ECF exhibits a pronounced increase in suitable habitat extent, whereas DBF shows only a modest expansion, particularly under the SSP1-2.6 scenario where the increase is minimal. These contrasting patterns highlight substantial differences in the magnitude of distributional shifts among forest community types, suggesting that aggregated forest metrics may mask important differences in vegetation-specific responses to climate change. Within the China Vegetation Classification System, vegetation formation represents one of the major hierarchical classification units and is defined based on integrated ecological conditions and physiognomic characteristics. Because these formations reflect long-term adaptations of terrestrial vegetation to specific environmental regimes (Guo et al., 2020; Chen et al., 2014), shifts at the vegetation formation level may signal fundamental changes in ecosystem structure and function rather than simple areal expansion.

Under the overall trend of warming and humidification across the QTP (Chen et al., 2015; Duan and Xiao, 2015), the northern Plateau has experienced particularly pronounced temperature increases since 1961 (Guo and Wang, 2012). ECF in this region occupies the high-elevation zone of the montane forest belt, and it is generally recognized that temperature is a key environmental factor limiting its distribution (Zhang et al., 2023a; Yin et al., 2023). Rising temperatures may alleviate thermal constraints, thereby facilitating the distribution of ECF. This interpretation is also supported by studies from the southeastern margin of the QTP, where rising temperatures have been shown to promote forest growth in high-elevation cold regions, particularly for treeline-dominant species such as *Picea, Abies* and *Juniperus* in the Hengduan Mountains (Shi et al., 2021). In contrast, DBF mainly occurs at lower elevations, where it is more strongly influenced by anthropogenic disturbances, resulting in more fragmented habitats and a relatively smaller distribution range. Although environmental conditions at lower elevations are generally more favorable than those at higher elevations in mountainous regions, forest distribution is not solely determined by abiotic factors. It may also be influenced by more complex processes such as interspecific competition and human disturbances (Huang et al., 2025; Luo et al., 2019). Therefore, under climate scenarios, the increase in suitable habitat for DBF is expected to remain limited.

Meanwhile, the magnitude and spatial pattern of expansion vary between scenarios, highlighting the importance of emission pathways in shaping future vegetation dynamics. Under the high-emission SSP3-7.0 scenario, forest suitable habitat expands more markedly than under SSP1-2.6, suggesting a nonlinear response of vegetation suitability to stronger warming. This may be attributed to the alleviation of extreme cold limitations, which currently restrict forest distribution at higher elevations and latitudes. Nevertheless, despite the projected increase in total suitable area, forest expansion remains spatially heterogeneous and is likely constrained by topographic complexity and moisture variability, which continue to exert fine-scale limitations.

### 4.3 Spatial shifts of suitable habitat distribution

Consistent with the redistribution patterns identified in the results, forest distributions in the region are projected to become increasingly concentrated in the eastern Qilian Mountains and the northern Gannan Plateau, although notable differences occur among vegetation types (**Figure 5**). This spatial redistribution of forest habitats results from the combined effects of multiple environmental factors, driven by the interaction between regional climatic conditions and topography–induced local microclimates (**Table 3**). It also reflects differences in the adaptive capacity of different plant functional types under future environmental changes (Li et al., 2026).

For ECF, although warming may theoretically alleviate thermal limitations, sustained temperature increases are accompanied by enhanced evapotranspiration, which can increase atmospheric water demand and accelerate soil moisture depletion. The northern slopes of the Qilian Mountains are particularly sensitive to this energy–water trade-off (Guo and Wu, 2025; Ma et al., 2024). Under ongoing climate change, intensified evapotranspiration on the northern slopes of the Qilian Mountains may aggravate regional aridity, progressively weakening the relative importance of temperature as a limiting factor and shifting the primary constraint toward moisture availability. This suggests that the potential thermal benefits of warming for high-elevation coniferous forest may be partially offset by increasing drought stress, resulting in a more complex and nonlinear response of ECF distribution than expected under a temperature-only control framework.

In contrast, DBF at lower elevations on the southern Gannan Plateau is mainly constrained by drought conditions (Yin et al., 2023), indicating a fundamentally different limiting mechanism compared with high-elevation coniferous forest. Although low-elevation environments are generally characterized by relatively higher energy availability, water deficit and seasonal drought stress remain the dominant ecological filters for deciduous broadleaf forest. As aridity is projected to intensify in the future, and the southern Gannan Plateau exhibits a consistent trend toward warming and drying (Man et al., 2025; Zhang et al., 2023b), increasing moisture limitation is likely to further restrict DBF persistence in its current range. Meanwhile, spatial heterogeneity in precipitation and topographic redistribution of moisture may promote a gradual shift of suitable habitats toward relatively wetter microclimatic zones, particularly in northern and more humid transitional areas. However, such redistribution is expected to be constrained and fragmented, given the combined influences of drought stress, habitat discontinuity, and potential biotic interactions, ultimately limiting the magnitude of DBF range expansion under climate scenarios.

### 4.4 Mechanisms underlying spatial redistribution

The spatial redistribution of forest vegetation in the study area is primarily driven by the interacting effects of moisture availability, thermal constraints, and topographic heterogeneity (**Table 3**). Rather than being controlled by a single dominant factor, forest distribution reflects a hierarchical filtering process in which large-scale climatic gradients define the broad distributional patterns, while local terrain conditions further refine habitat suitability and spatial configuration.

At the forest vegetation formation group level, precipitation-related variables emerge as the primary climatic control, indicating that water availability is the fundamental limiting factor for forest establishment in this cold and semi-arid mountainous region. The strong influence of precipitation seasonality further suggests that not only total water input but also its intra-annual distribution plays a critical role in shaping forest persistence. In ecosystems characterized by pronounced seasonal aridity, temporal water stress can be as limiting as absolute moisture deficiency, particularly during the growing season when water demand peaks. These patterns are consistent with previous findings emphasizing the dominant role of hydroclimatic constraints in shaping vegetation patterns in arid and semi-arid mountain ecosystems (Yang et al., 2014; Wang et al., 2020). In addition to moisture constraints, temperature exerts a persistent limiting effect. This is particularly relevant in high-elevation environments where growing season length is shortened and frost-related stress is frequent. The combined influence of moisture limitation and thermal restriction thus defines a dual climatic constraint shaping forest distribution across both elevational and longitudinal gradients in the region (Sherry et al., 2012; Chen et al., 2022). Along elevational gradients, decreasing temperature imposes strong physiological constraints, whereas along longitudinal gradients, increasing aridity becomes the primary limiting factor, jointly structuring the regional distributional boundary of forests.

At the vegetation formation level, ECF exhibits a relatively balanced response to moisture and temperature conditions, reflecting its broad ecological tolerance and strong adaptability to cold and relatively dry environments. Its distribution is therefore closely linked to stable water supply as well as resistance to low-temperature stress, enabling it to occupy a wide range of mountain habitats. This pattern is consistent with previous studies showing that coniferous forests in high-elevation regions are jointly regulated by moisture availability and cold-season temperature constraints (Pu et al., 2025; Ma et al., 2022). DBF is more strongly constrained by temperature extremes, particularly low winter temperatures, indicating a narrower climatic niche and greater sensitivity to thermal thresholds. This suggests that DBF distribution is primarily governed by physiological limitations related to cold stress, which restricts its occurrence in high-elevation and more thermally unstable environments.

Although terrain factors exhibit relatively lower importance compared with climatic variables, they still play a meaningful role in modulating forest distribution patterns. Elevation, in particular, contributes to shaping spatial heterogeneity by influencing temperature regimes, moisture redistribution, and energy balance (Zhao et al., 2022). The steep elevational gradients in the study region generate rapid climatic transitions over short distances, thereby amplifying local environmental heterogeneity and reinforcing spatial segregation among forest types.

## 5 Limitations and future directions

This study projects climatically suitable habitats primarily based on climatic and terrain variables; however, several sources of uncertainty should be acknowledged when interpreting the results. First, the RF model assumes unlimited dispersal and does not explicitly incorporate factors such as dispersal limitations, soil properties, disturbance regimes, or biotic interactions, all of which may influence realized forest distributions. Second, potential spatial biases associated with background point selection and presence-only data may also affect model outputs, despite efforts to ensure ecological representativeness. Future studies could improve projection robustness by explicitly incorporating ecological processes and disturbance dynamics, thereby providing a more comprehensive understanding of forest adaptive capacity under climate change.

## 6 Conclusions

Based on RF models, this study projected changes in the climatically suitable distributions of forest community types along the northeastern margin of the QTP under climate scenarios. Under both the sustainability scenario (SSP1-2.6) and the regional rivalry scenario (SSP3-7.0), the total area of climatically suitable forest habitats is projected to increase substantially, resulting in an approximately twofold expansion compared with the current period. This expansion is primarily driven by ECF, which exhibits the most pronounced increase in suitable habitat, whereas the expansion of DBF remains relatively limited. In addition to changes in habitat extent, the spatial distribution of suitable habitats is projected to shift considerably. The eastern Qilian Mountains and northern Gannan Plateau are identified as key hotspots for future forest expansion, where climatic conditions are expected to become increasingly favorable for forest establishment and persistence. In contrast, areas currently suitable for ECF on the northern slopes of the Qilian Mountains, as well as regions supporting DBF in the southern mountainous areas of the Gannan Plateau, are projected to become unsuitable under climate conditions, indicating potential redistribution of forest community along regional environmental gradients. The projected shifts highlight the importance of considering climate-driven redistribution patterns in future forest management, conservation planning, and ecological restoration strategies. Importantly, our results show that different vegetation types respond differently to climate change, emphasizing the need to distinguish among forest types in ecological assessment and management. Overall, these findings provide valuable scientific insights for enhancing the resilience of forest ecosystems in the northeastern QTP under future climate change.

## Data Availability

The raw data supporting the conclusions of this article will be made available by the authors, without undue reservation.
